# Changes in Volatile Composition of Cape Hake Fillets under Modified Atmosphere Packaging Systems during Cold Storage

**DOI:** 10.3390/foods11091292

**Published:** 2022-04-29

**Authors:** Umezuruike Linus Opara, Tobi Fadiji, Oluwafemi James Caleb, Adebanji Olasupo Oluwole

**Affiliations:** 1 SARChI Postharvest Technology Research Laboratory, Africa Institute for Postharvest Technology, Faculty of AgriSciences, Stellenbosch University, Stellenbosch 7602, South Africa; fadiji@sun.ac.za (T.F.); caleboj@sun.ac.za (O.J.C.); astsupo@yahoo.com (A.O.O.); 2 UNESCO International Centre for Biotechnology, Nsukka 410001, Enugu State, Nigeria; 3 Department of Food Science, Faculty of AgriSciences, Stellenbosch University, Stellenbosch 7602, South Africa

**Keywords:** cape hake, fish fillets, modified-atmosphere packaging, volatile composition, storage temperature

## Abstract

Fresh ready-to-cook fish fillets are susceptible to loss of freshness and accumulation of off-odour due to accelerated microbial spoilage. Suboptimal storage temperature and packaging conditions accelerate this process, limiting the economic potential. This study investigated the effects of modified atmosphere packaging (MAP) and storage temperature (0 °C and 4 °C) on the volatile compounds (VOCs) of Cape hake (*Merluccius capensis*) fish fillets as a predictor of shelf life and quality. Fresh Cape hake fillets were packaged under active modified atmosphere (40% CO_2_ + 30% O_2_ + 30% N_2_) and passive modified atmosphere (0.039% CO_2_ + 20.95% O_2_ + 78% N_2_) with or without an absorbent pad and stored at 0 °C and 4 °C for 12 d. The results obtained demonstrated that changes in VOCs and concentration were significantly (*p* < 0.05) influenced by MAP conditions, storage temperature and duration. A total of 16 volatiles were identified in the packaged Cape hake fillets: 4 primary VOCs and 12 secondary VOCs. The spoilage VOCs identified include tri-methylamine (TMA) (ammonia-like), esters (sickeningly sweet) and sulphur group (putrid). The concentration of secondary VOCs increased continuously during storage. Active-MA-packaged fillets performed better and had lower TMA values of 0.31% at 0 °C on day 12 in comparison to 7.22% at 0 °C under passive on day 6. Ethyl acetate was detected in passive-MA-packaged fillets stored at 0 °C on day 3, and the levels increased to 3.26% on day 6, while active-MA-packaged fillets maintained freshness. This study showed that in conjunction with TMA, VOCs such as esters and sulphur-related compounds could be used as spoilage markers for Cape hake fish fillets.

## 1. Introduction

Fish provides about 17% of the world’s annual protein intake [1]. Moreover, it is also a healthy alternative to meat due to the presence of poly-unsaturated fatty acids, which increase fish’s nutritional value, and guard against disease conditions, such as high blood pressure and cholesterol levels in humans [1,2,3,4,5,6]. Fresh fish is highly perishable due to its high water activity (aw ≥ 0.95) and post-mortem pH level of about 5.2 [7,8,9]. The quality of fresh fish and fishery products has been assessed by measuring chemical, microbial, physical and sensory attributes [10,11,12]. Although sensory attributes are vital in determining fish wholesomeness, other fast and unbiased ways to support recommendations of sensory panels are needed [10,13].

Change in quality attributes of fresh fish arises because of fish tissue degradation and changes in volatile compounds (VOCs) that evolve from the fish. Studies have shown that spoilage occurs due to bacteria infestation in fish muscles which triggers a continuous breakdown of glycogens, nucleotides, amino acids and other non-protein nitrogen [11,14]. Spoilage results in the release of volatile compounds such as tri-methyl amine (TMA), aldehydes, ketones, esters, hypoxanthine and low-molecular-weight sulphur complexes, which contribute to the characteristic odour of spoilt fish [11,13,15]. Specific VOCs have been identified as vital descriptors of the aroma and freshness of fresh fish and fishery products [16,17,18].

Changes in VOCs before fish quality deterioration have been reported [10,17,19]. Other researchers have noted that these volatile compounds can be used to assess changes in the freshness of packaged fresh fish fillets during storage [10,13,20,21,22]. For example, Giogios et al. [21] reported that commercial fresh Mediterranean seafood comprising seven types of fish such as sand-smelt, picarel, hake, pilchard, bogue and anchovy, and crustaceans including striped-mullet, squid, shrimp and mussel, contained a total of 298 VOCs. Among these types of fish, pilchard had a higher number of VOCs, whereas mussels had the highest concentration of VOCs. Furthermore, levels of alcohols in pilchards were high; amines and sulphur VOCs were present in shrimps, while aldehydes, furans, pyridine, pyrazines and pyrrols were detected in mussels at elevated levels [21]. Boscaino et al. [22] compared the effects of vacuum packaging (VP) and home refrigeration at 4 °C on the quality attributes of rainbow trout for 6 days. They found that VP maintained the quality attributes of trout better than refrigerated storage by controlling oxidation and decay. Additionally, VP controlled foul aromas and improved flesh firmness with enhanced fish flesh colour and restricted odour development by day 6 [22].

Numerous researchers have reported the benefits of modified-atmosphere packaging (MAP) in extending the shelf life of fillets of different fish species [5,23,24,25,26,27,28,29]. Lauzon et al. [26] found that fillets in MAP (50% CO_2_ + 5% O_2_ + 45%) had a higher shelf life of about 15 d at 0 °C and 21 d at −2 °C, compared with 11 d at 0 °C and 14 d at −2 °C in normal-air packaging. Similarly, a study investigating the role of active-MAP [(A) = 40% CO_2_ + 30% O_2_ + 30% (B) = 50% CO_2_ + 50% O_2_ and (C) = 95% CO_2_ + 0% O_2_], combined with a mixture of three natural preservatives (thymol, lemon extract and grapefruit seed extract) on bluefish burger (mackerel and hake) stored at 4 °C for 28 d was reported by Del Nobile et al. [5] who found lower aerobic mesophilic bacteria counts in active-MAP fillets. In the study by Bouletis et al. [30], the authors found that cuttlefish shelf life reached 2, 2, 4, 8 and 8 days for control, VP, MAP 1, MAP 2 and MAP 3 (20% CO_2_-80% N_2_, 50% CO_2_-50% N_2_ and 70% CO_2_-30% N_2_ for MAP 1, 2 and 3, respectively).

Cape hake is an important seafood in South Africa and falls under wild capture fisheries. Although Cape hake accounts for roughly 80% of the total economic contribution of the fish industry to the economy, the product’s short shelf life and poor quality remain significant challenges. Furthermore, one of the most commonly traded fish fillets in South Africa is Cape hake fillets, which are economically important to South African fish industries and are frequently found in retail outlets. Additionally, due to the ease of packaging and consumer acceptance, Cape hake is sold in fillets. To the best of our knowledge, these previous studies did not consider the changes in VOCs as a potential descriptor of postharvest quality of packaged fresh fish fillets under MAP. Hence, the objective of this study was to investigate the effects of MAP, combined with absorbent pads and storage temperature, on the changes in volatile compounds and concentration of Cape hake fish fillets.

## 2. Materials and Methods

### 2.1. Preparation of Fish Samples and Packaging

Fresh Cape hake fillets (average weight of 200 g) were purchased from a local retail outlet in Stellenbosch, South Africa. Fillets were iced with the appropriate quantity of ice (with 1:3 parts *w/w* flake/ice) and packed in a sterile padded polystyrene box. Fillets were collected approximately 18 h after preparation and conveyed to the postharvest laboratory within 15 min. On arrival, the bulk fish fillets were divided into four batches representing the individual treatments considered in this study. The fish fillets were kept on ice and packaged into the following treatments: active-MA (40% CO_2_ + 30% O_2_ + 30% N_2_) without absorbent pad; (40% CO_2_ + 30% O_2_ + 30% N_2_) with absorbent pad; passive-MA (0.039% CO_2_ + 20.95% O_2_ + 78% N_2_) (PMAP) with absorbent pad; and (0.039% + 20.95% + 78%) without absorbent pad (as control).

**Figure 1:**
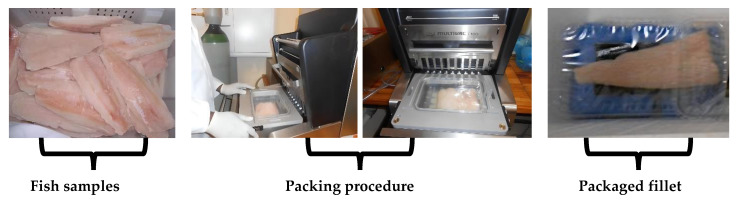
An illustration of the fish fillet packaging approach.

All fish samples were packaged in polyethylene terephthalate (PET) trays with dimensions 280 × 190 mm (Zibo containers (Pty.) Ltd., Kuilsrivier, Cape Town, South Africa) and heat-sealed with bi-axially oriented polyester film (O_2_ permeability: 75 cm^3^ at 23 °C, 70% RH bar^−1^; water vapour permeability: 2 g·d^−1^ at 38 °C, 90% RH) from Knilam Packaging (Pty.) Ltd., Cape Town, South Africa. A total of 380 packages were used in this study. Modified-atmosphere packaging was performed using a Multivac packaging machine (Multivac Traysealer T100, Sepp Hagenuller GmbH & Co.KG, Wolfertschwenden, Germany), and food-graded gases were obtained from Air products Pty., Kempton Park, South Africa, as well as absorbent pads (Dri-Fresh^®^, Sirane Ltd., Telford, Shropshire, UK). According to the manufacturer’s specification, the absorbent pads were made of inert hygroscopic material. Packaged fillets were then stored for 15 d at 0 °C and 4 °C. Three fish packs were taken for each treatment and temperature on each sampling day. Sampling was done on days 0, 3, 6, 9, and 12. Figure 1 depicts the fish samples, packing procedure, and packaged fish fillet.

### 2.2. Extraction of Volatile Compounds and Chromatographic Analysis

For each packaged fish fillet, approximately 7 g of fish fillets was taken and placed in a 20 mL solid-phase micro-extraction (SPME) vial containing 3 g of NaCl [31]. To each SPME vial with the fish sample, 5 mL of double distilled water and 50 μL of 3-Octanol (internal standard) were added, and the vial was gently vortexed to mix the contents. Trapped volatile compounds (VOCs) in the vial headspaces were then extracted on a SPME fiber. The vials were permitted to equilibrate for 2 min at 50 °C in the CTC autosampler incubator (with an agitation speed of 250 rpm). A 50/30 μm fibre coated with divinylbenzene/-carboxen/-poly-dimethylsiloxane was inserted (10 mm deep) into the vial headspace and exposed for 10 min at 50 °C [31]. For desorption of VOCs, the fibre coating was injected into the gas chromatography–mass spectrometry (GC–MS) for 2 min in splitless mode. The fibre was inserted in a fibre conditioning station for 15 min between samples for cleaning to prevent cross-contamination [31]. The temperature of the gas chromatography (GC) injection port was maintained at 250 °C. The oven temperature program was as follows: the oven temperature was maintained at 70 °C for 1 min, and then ramped up to 142 °C at the rate of 3 °C/min; then finally ramped up to 240 °C at the rate of 5 °C/min and kept at that temperature for 3 min [31,32].

Separation of VOCs was performed using GC (Agilent 6890 N, Agilent, Palo Alto, CA, USA), coupled with a mass spectrometer detector (Agilent 5975 MS, Agilent, Palo Alto, CA, USA) [31]. The GC–MS system was equipped with a polar DB-FFAP column (Model number: J&W 122-3263), with a nominal length of 60 m, 250 μm internal diameter, and 0.5 μm film thickness. Analyses were carried out using helium as carrier gas at a constant flow rate of 1.9 mL/min [31]. The MS data were collected on MSD operated in full scan mode, and the ion source and quadrupole temperatures were maintained at 230 °C and 150 °C, respectively. The MSD transfer line was held at 280 °C. The individual peaks were categorised by comparing their retention times (RT) and with mass spectral library (NIST, ver. 2.0) and certified with the database Flavornet in conjunction with those published in the literature [32,33,34].

### 2.3. Microbiological Analysis

Approximately 1 g of fish sample was taken from randomly selected packaged fillets for each treatment on the sampling day and mashed using mortar and pestle under aseptic conditions. The mashed sample was then diluted in test tubes containing 10 mL of sterile physiological saline solution (PSS) (0.85 g NaCl in 100 mL distilled water). Serial dilutions up to four-fold were prepared by adding 1 mL of homogenate sample to 9 mL PSS and vortexing each dilution. An amount of 1 mL of each dilution was plated onto appropriate media in triplicate using the pour plate method to count the microbial load. [35].

Aerobic mesophilic bacteria were counted using plate count agar (PCA) method 4833 [36]. The plates were incubated upside down at 37 °C for 48 h [37]. For the screening of *Escherichia coli*, violet-red bile glucose (VRBG) agar was used, and plates were incubated upside down at 37 °C for 24 h [38]. The presence of *Vibrio parahaemolyticus* in fish fillet was investigated using a combination of thio-sulphate bile salt sucrose agar (TCBS) and HiChrome *vibrio* agar, method 21872-1 [39]. HiChrome *vibrio* Agar was used because colour development by *Vibrio* species is not affected by the presence of colonies of other bacteria as the amount of colour developed depends on the reaction of bacterial ß-galactosidase with the substrate contained in the media. The plates were then incubated upside down at 37 °C for 24 h [37]. After incubation, plates with 30–300 colonies were counted. The results were transformed into Log colony forming unit (log CFU g^−1^).

### 2.4. Statistical Analysis

Statistical analysis was carried out using Statistica software (Statistica version 11, StatSoft Inc., Tulsa, OK, USA). Analysis of variance (ANOVA) was used to evaluate the effect of modified-atmosphere packaging (MAP) on the volatile quality of packaged Cape hake fillets. The variation between the mean values was examined according to Duncan’s multiple range tests at *p* < 0.05. To establish correlation trends between microbial count and volatile quality of the examined Cape hake fillets, data were subjected to Pearson’s correlation coefficient using XLSTAT software Version 2012.4.01 (Addinsoft, Paris, France). Significant correlation coefficients were classified as strong, and moderate corresponding to r > 0.7 and r > 0.5 −< 0.7, respectively.

## 3. Results and Discussion

### 3.1. Microbiology Analysis

Fillets used in this study had good microbial quality, as evidenced by low initial aerobic mesophilic bacteria (1.2 log·cfu/g) on fresh samples. Furthermore, it is worth mentioning that in this study, both *Escherichia coli* and *Vibrio parahaemolyticus* were not detected in any of the samples.

The lowest aerobic mesophilic bacteria were observed in fillets packaged under active-MAP and stored at 0 °C, while passive-MAP-stored fillets stored at 0 °C had the highest (7.13 log cfu/g by day 6) without absorbent pads (Table 1). In fillets stored at 0 °C under passive-MA, aerobic mesophilic bacteria count reached the critical limits of <5.5 log cfu/g by day 6 [38,39]. For fillets under active-MAP storage at 0 °C, AMCs did not exceed the critical limit until after day 12 and at 4 °C, reaching 6.0 and 7.2 log cfu/g for 9 and 12 d, respectively (Table 1). These results agreed with the findings of Ordonez et al. [23] for MAP conditions (40% CO_2_ + 60% air, 20% CO_2_ + 80% air and 100% passive-MAP) for hake fillets stored at 2 °C for 12 d. They reported that AMCs exceeded the microbial limits faster in fillets stored under 100% passive-MAP fillets than in active-MAP-stored fillets. Additionally, MAP (40% CO_2_ + 60% air) was more efficient in hindering microbes. This highlights MAP’s value addition in maintaining shelf life and quality of packaged fresh ready-to-cook fish products.

Furthermore, absorbent pads had a significant impact on the microbial load of fillets (*p* < 0.05). Lower microbial counts were observed for active-MA-packaged fillets with absorbent pads stored at 0 °C and 4 °C, but the use of the pad was only effective at 0 °C for passive-MA-packed fillets (Table 1). The lower microbial growth observed at 0 °C during storage might be due to the increased solubility of CO_2_ at lower temperatures, which increases the acidity of fish tissue and inhibits microbial growth. Higher temperatures inevitably result in less dissolved CO_2_ in the product and, as a result, a loss of inhibitory effect, resulting in increased microbial and enzymatic activity. Hence, the rate of deterioration is highly temperature-dependent and can be slowed by using a low storage temperature [25]. Similar results were reported by Ordonez et al. [23] for hake steaks and chub mackerel by Stamatis and Arkoudelos [40]. The authors stated that aerobic mesophilic bacteria were best inhibited at lower storage temperatures due to increased solubility of CO_2_.

Furthermore, it is worth noting that the observed decrease in microbial growth could be due to changes in atmospheric conditions. Active MAP suppressed the growth of aerobic bacteria. This can be attributed to lag phase extension of the spoilage bacteria [5].

### 3.2. Volatile Composition

A total of 16 VOCs were identified from MA-packaged Cape hake fillets. This includes 4 primary VOCs identified on the fresh fish sample and 12 secondary VOCs that evolved with storage duration (Table 2 and Table 3). The volatile compounds found in Cape hake fish fillets were grouped into six chemical classes, namely: (a) alcohols; (b) Ketones; (c) organic acid; (d) amines; (e) esters; and (f) sulphur-containing compounds (Table 2). Similar classes of VOCs have been reported for fish such as cod gilthead sea bream, sand-smelt, picarel, hake, pilchard, bogue and anchovy and fishery products such as squid, shrimp and mussel [10,21,22,42]. Temperature, modified atmosphere and storage duration had a significant impact (*p* < 0.05) on change in composition and the relative abundance of VOCs in packaged fillets. For example, there was an initial increase in the levels of ethyl alcohol across all treatments, but this decreased as storage progressed (Table 3). These results were consistent with other reports on the volatile composition in cod fish [42]. Although the levels of ethyl alcohol increased initially, the authors stated that there was a subsequent decrease as storage progressed and concluded that the volatile quality of cod fish filets was affected by temperature and storage time [42]. Additionally, the initial high levels in ethyl alcohol could be because of the fermentation of glycogen and its breakdown by lactic acid bacteria [43].

VOCs increased as storage temperature increased from 0 °C to 4 °C on each measurement day (*p* < 0.05). For instance, lower concentrations of 3-methyl-1-butanol were observed in active-MAP stored fillets at 0 °C when compared with 4 °C; the values were 0.33% and 0.54%, respectively, on day 6 (Table 3). Similar results were reported by Edirisinghe et al. [44] in their study, which investigated VOCs in yellow fin tuna at 0 °C and 30 °C using SPME and GC–MS. The authors found that the levels of 3-methyl-2-butanol were higher in tuna stored at 30 °C than 0 °C. Furthermore, 3-methyl-2-butanol was detected after 24–36 h storage in fillets stored at 30 °C and on day 16 at 0 °C [44]. Similarly, Olafsdottir et al. [45] investigated the effects of storage temperature on decay microbes using an electronic nose in haddock fillets packaged in expanded polystyrene boxes and stored at 0 °C, 7 °C and 15 °C. The results showed that decay alcohols, aldehydes, esters, amine and sulphur volatiles were detected between day 2 and 3 at 15 °C, between day 2 and 7 for fillets stored at 7 °C, and days 4 and 9 for fillets stored at 0 °C. Furthermore, the concentrations of these decay volatiles were highest in fillets stored at 15 °C and lowest at 0 °C (*p* < 0.05) [45].

Active-MAP maintained the evolution and composition of VOCs better than passive-MAP (Table 3). Production of TMA was significantly lower in active-MA-packaged fillets throughout the storage period and across temperatures (Table 3). In passive-MAP fillets, TMA were detected on day 3 and increased throughout storage, ranging from 0.25% in fillets stored in active-MAP at 0 °C on day 12 to 7.22% in fillets packaged in passive-MAP at 0 °C on day 6. Similar results were reported by Alasalvar et al. [46] in their study comparing the VOCs in farmed and wild sea bream stored at 2 °C and 4 °C for 23 days. The authors reported that TMA levels increased in both fish fillets during storage and suggested that this could result from bacterial degradation of marine fish fillets. Other studies have indicated that TMA may be used as an index of microbial deterioration because Gram-negative microbes acquire energy by reducing tri-methyl amine oxide to TMA, thereby creating fishy odours at low levels and ammonia-like odours at higher levels [15,41]. High levels of TMA in fish lead to spoilage due to short shelf life and low fillet quality [42].

Other spoilage indicator volatile groups identified in the present study include acetic acid, phenyl ethyl alcohol, butylated hydroxy toluene, butane 1, 2-diol, esters and sulphur (Table 3). Acetic acid was detected in passive-MA-packaged fillets stored at 0 °C on day 3, and this compound increased continuously with the storage duration; the value on day 6 was 0.35% (Table 3). Studies have indicated that the formation of organic acids might be due to bacterial fermentation of amino acids and lipid oxidation, resulting in acid production [46]. Acetic acid and other organic acids were identified as the source of the unpleasant odours during the storage of herring at 6 °C for 8 days [46,47].

Similarly, butane 1, 2-diol increased from 0.07% to 0.1% in passive-MA-packaged fillets at sensory rejection on day 6 (Table 3). Likewise, esters were detected in passive-MA-packaged fillets on day 3 for fillets stored at 0 °C, and the value for ethyl acetate was 3.26% on day 6 when they were rejected (Table 3). In contrast, these VOCs were not detected in active-MA fillets stored at 0 °C at the end of storage as active-MAP and the lower temperature helped maintain volatile quality. Furthermore, the presence of esters could be due to microbial activity. Similar observations were reported in a study that characterised the volatiles in chilled cod fillets stored at 0.5 °C, using GC and an electronic nose [42]. The authors noted that esters and sulphur compounds were detected at the spoilage stage on day 17 [42]. In addition, di-methyl sulphur was detected in passive-MA-packaged fillets stored at 0 °C on day 6 (2.9%). In comparison, sulphur spoilage volatiles were not detected in active-MA-packaged fillets stored at 0 °C at the end of storage (Table 3). Similar results were obtained with studies on the VOCs of cod using GC and electronic nose at 0 °C and abuse temperatures [42].

The level of VOCs was higher in packages without pads than those with pads, which was consistent for all temperature conditions (Table 3). For instance, the concentration of 3-methyl-1-butanol in packages without pads was higher than in those with pads. The active-MA-packaged fillet without pads was 0.74%, while packages with pads had concentrations of 0.54% at 0 °C. Absorbent pads helped reduce the levels of secondary VOCs such as esters and sulphur volatiles during storage across all treatments. For example, ethyl acetate levels in passive-MAP without pads were 3.26%, while packages with pads had concentrations of 3.09% at 0 °C. In the same vein, dimethyl sulphide levels in passive-MAP without pads were 2.86%, while the package with pads had concentrations of 2.22% at 0 °C. Similar results were obtained by Olafsdottir et al. [42] in a study that classified VOCs present in frozen cod fillets using GC and electronic nose stored in expanded polystyrene boxes at 0.5 °C. The authors proposed that the dimethyl sulphide, dimethyl disulphide and other sulphur VOCs were decay markers in cod and were the origin of putrid odours. However, improper storage protocols could lead to the evolution of dimethyl trisulphide in fresh cod [16,42]. Sulphur VOCs are formed when microbes degrade sulphur-containing amino acids in fish, such as cysteine and methionine. Further oxidation leads to the formation of dimethyl sulphide and dimethyl disulphide [16].

Furthermore, Hansen et al. [48] investigated the chemical and microbial quality of dairy farm red smear cheese made from pasteurized and unpasteurized milk studies. The authors reported that late acidification of red smear cheese influenced the production of VOCs such as dimethyl sulphide, which could be attributed to higher microbial counts. Furthermore, they concluded that lower pH resulted in lower microbial counts in red smear cheese [48]. Results from this study are consistent with findings in the literature that higher CO_2_ levels better maintain the volatile quality of muscle meat at 0 °C and 4 °C, with 0 °C being the optimum storage temperature [20].

### 3.3. Correlation between Quality Indices

Pearson’s correlation was conducted between aerobic mesophilic counts and selected spoilage indicators in the VOCs to identify spoilage markers that predict Cape hake fish’s shelf life and quality attributes (Table 4). Significant associations of interest with various correlation coefficients were obtained. Moderately positive relationships (r = 0.57) and (r = 0.66) were established between aerobic mesophilic counts (AMCs) and ethyl acetate and butanoic acid esters, suggesting that the presence of moderate amounts of esters in the headspace of fish fillets could have been produced by the AMCs (Table 4). In addition, a moderate association was established between AMCs and di-methyl sulphide (r = 0.68); in contrast, strong correlations were shown between AMCs and dimethyl disulphide (r = 0.73). This implies that sulphur-containing volatiles could be good spoilage indicators in Cape hake fish fillets. The use of SPME/GC/MS has led to the establishment of correlations between AMCs and dimethyl sulphide in fresh and iced fish such as European sea bass, gilthead sea bream, cod and salmon. However, correlations between AMCs and dimethyl disulphide have only been established in red smear cheese [48,49].

Furthermore, moderate correlations were observed between AMCs and TMA (r = 0.50), implying that TMA might not be a good spoilage marker as spoilage might have occurred before TMA was detected in the headspace of packaged fillets. Nevertheless, strong positive correlations were established between TMA and other VOCs; the values were ethyl acetate (r = 0.84), butanoic acid ester (r = 0.88), di-methyl sulphide (r = 0.80) and dimethyl disulphide (r = 0.74). This suggests that TMA could be used in conjunction with other spoilage markers to predict the shelf life and quality of Cape hake fish fillets during storage.

## 4. Conclusions

This study investigated the effects of MAP with/without absorbent pads on the VOCs of packaged Cape hake fillets at 0 °C and 4 °C. The use of MAP with absorbent pads significantly impacted changes in volatile compounds of Cape hake fillets at 0 °C and 4 °C during storage (*p* < 0.05). Comparison of the changes in the volatile compositions during storage and their correlations with AMCs led to the identification of spoilage compounds that could be used to predict shelf life and quality indicators for packaged Cape hake fish fillets. Fillets in active-MAP packages outperformed passive-MA-packaged fillets stored at 0 °C, as evidenced by lower levels of TMA, ester, and sulphur groups. Based on measured microbial and volatile attributes, active-MAP (40% CO_2_ + 30% O_2_ + 30% N_2_) combined with low-temperature storage (0 °C) extended the shelf life and maintained the quality attributes of Cape hake fillets for 12 d. These findings provide a useful guide in fresh fish processing towards predicting the shelf life and quality of Cape hake fillets and other fish products during storage. Rapid detection and monitoring of the concentrations of spoilage VOCs such as esters, in conjunction with tri-methyl amine, detected early during storage, can provide warning signals (spoilage markers) on the quality and safety status of fresh Cape hake during postharvest handling and fillet marketing. Future studies will consider developing a model to predict the shelf life of the Cape hake fillets using an integrated and multiple perspectives (chemical, microbiological, physical, and sensory) approach.

## Figures and Tables

**Table 1:** Effects of packaging with/without absorbent pad, temperature (0 °C and 4 °C) and storage time (d) on bacterial growth of Cape hake fillet.

STORAGE DAYS	TEMP. (°C)	MAP − PAD	MAP + PAD	PMAP − PAD	PMAP + PAD
0		1.2 ± 0.13 ^j^	1.2 ± 0.13 ^j^	1.2 ± 0.13 ^j^	1.2 ± 0.13 ^j^
3	0	3.6 ± 0.07 ^i^	3.5 ± 0.05 ^i^	3.9 ± 0.05 ^g^	3.7 ± 0.09 ^h^
	4	3.8 ± 0.04 ^h^	3.6 ± 0.01 ^h^	6.2 ± 0.09 ^c^	6.2 ± 0.03 ^c^
6	0	4.5 ± 0.04 ^f^	4.5 ± 0.04 ^f^	7.1 ± 0.1 ^a^	6.9 ± 0.03 ^a^
	4	4.8 ± 0.03 ^e^	4.8 ± 0.03 ^e^	nd	nd
9	0	5.0 ± 0.03 ^e^	4.9 ± 0.03 ^e^	nd	nd
	4	6.0 ± 0.03 ^d^	5.9 ± 0.03 ^d^	nd	nd
12	0	5.2 ± 0.03 ^e^	5.1 ± 0.03 ^e^	nd	nd
	4	7.2 ± 0.03 ^a^	7.2 ± 0.03 ^a^	nd	nd

nd = Not determined due to sensory rejection. Different letters indicate significant differences in pH values (*p* < 0.05) according to Duncan’s Multiple Range tests; MAP − PAD: active-MA without absorbent pad; MAP + PAD: active-MA with absorbent pad, PMAP − PAD: passive-MA without absorbent pad and PMAP + PAD: passive-MA with absorbent pad. Sampling was stopped on days when bacterial growth exceeded the microbial limit of <5.5 log cfu/g by day 6 [38,39]. Thus, sampling for fillets stored under PMAP at 4 °C was stopped on day 3; furthermore, sampling under PMAP at 0 °C was stopped on day 6. Research findings have indicated that CO_2_ retards the growth of psychotropic, aerobic and Gram-negative microbes and slows down the deterioration of fresh RTC fish fillets [40]. Delayed deterioration in active-MAP stored fillets because dissolved CO_2_ leads to the formation of carbonic acid. The un-dissociated form of carbonic acid (bicarbonate ion) changes cell permeability and hinders microbes’ metabolic processes [41]. Therefore, active-MAP combined with an absorbent pad at optimum cold storage (0 °C) can assist in extending the shelf life and maintaining microbial safety of RTC hake fillets.

**Table 2:** Volatile compounds identified from MA-packaged Cape hake fillets.

Compound	RT (min)	Similarity (%)	Aroma Descriptor ***
*Alcohol*			
** Ethyl alcohol	4.73	91	Sweet
** 3-methyl-1-butanol	10.4	86	Malt
** 2-ethyl-1-hexanol	20.9	83	Sweet
1,2-butanediol	7.74	83	Unknown
Phenyl ethyl alcohol	35.1383	97	Spicy
Butylated hydroxy toluene	34.8562	95	Spicy, phenolic
*Ketone*			
** 3-octanone	12.3639	83	Earthy, mushroom
2,3-butanedione	5.3933	53	Butter, cheese
4-hydroxy-4 methyl-2 pentanone	16.5687	78	Unknown
*Organic acid*			
Acetic acid	19.92	58	Sour
*Amine*			
Tri-methylamine	3.7916	72	Fish-like
*Ester*			
Ethyl acetate	4.349	72	Pineapple
Butanoic acid, ethyl ester	6.3173	91	Fruity, banana
*Sulphur*			
1-Propanol, 3-(methyl thio)-	29.7057	99	Unknown
Dimethyl sulphide	3.566	94	Cabbage
Dimethyl disulphide	7.3122	94	Onion, putrid

RT = retention time; (MS software, NIST version 2.0); ** = primary volatile compounds; *** [32,33,34].

**Table 3:** Common volatiles identified from MA-packaged Cape hake fillets using gas chromatography–mass spectra analysis showing freshness and spoilage indicator markers expressed as % peak areas indicating sampling days 0, 6 and 12.

Common Volatiles	RT	Day 0			Day 6						Day 12	
	(Min)											
			0 °C MAP −	0 °CMAP +	0 °C PMAP	0 °C PMAP +	4 °CMAP −	4 °C MAP+	0 °C MAP −	0 °C MAP +	4 °C MAP −	4 °C MAP +
			PAD	PAD	PAD	PAD	PAD	PAD	PAD	PAD	PAD	PAD
Ethyl alcohol	4.73	0.6 ± 0.10 ^t^	2.3 ± 0.12 m^e^	2.2 ± 0.12 ^n^	3.6 ± 0.12 ^c^	3.2 ± 0.12 ^f^	3.0 ± 0.12 ^j^	2.9 ± 0.12 ^k^	1.1 ± 0.12 ^r^	0.7 ± 0.12 ^s^	2.2 ± 0.12 ^n^	2.1 ± 0.12 ^o^
3-methyl butanol	10.4	0.1 ± 0.03 ^q^	0.3 ± 0.04 ^l^	0.2 ± 0.01 ^n^	1.0 ± 0.03 ^c^	0.9 ± 0.02 ^d^	0.5 ± 0.01 ^h^	0.5 ± 0.01 ^h^	0.7 ± 0.01 ^f^	0.5 ± 0.01 ^h^	1.3 ± 0.03 ^a^	1.1 ± 0.01 ^b^
2-ethyl hexanol	20.9	0.3 ± 0.06 ^a^	nd	Nd	0.2 ± 0.02 ^b^	0.2 ± 0.02 ^c^	nd	Nd	nd	nd	nd	nd
3-octanone	12.36	0.2 ± 0.01 ^s^	0.4 ± 0.01 ^n^	0.3 ± 0.02 ^p^	1.0 ± 0.02 ^a^	0.9 ± 0.02 ^b^	0.5 ± 0.02 ^l^	0.4 ± 0.01 ^h^	0.8 ± 0.01 ^d^	0.55 ± 0.02 ^i^	0.88 ± 0.01 ^c^	0.8 ± 0.02 ^d^
Tri-methylamine	3.79	nd	nd	Nd	7.2 ± 0.07 ^a^	5.4 ± 0.05 ^b^	nd	Nd	0.3 ± 0.03 ^k^	0.1 ± 0.03 ^l^	2.4 ± 0.14 ^g^	1.8 ± 0.13 ^h^
Ethyl Acetate	4.35	nd	nd	Nd	3.3 ± 0.01 ^a^	3.1 ± 0.01 ^b^	nd	Nd	nd	nd	1.2 ± 0.01 ^c^	1.2 ± 0.01 ^d^
Butanoic acid Ester	6.32	nd	nd	Nd	0.8 ± 0.002 ^a^	0.6 ± 0.001 ^b^	nd	Nd	nd	nd	0.3 ± 0.001 ^c^	0.2 ± 0.001 ^d^
Acetic acid	19.92	nd	nd	Nd	0.4 ± 0.002 ^a^	0.4 ± 0.002 ^b^	nd	Nd	nd	nd	0.2 ± 0.007 ^c^	0.1 ± 0.002 ^d^
3-methyl thio-1-propanol	29.71	nd	nd	Nd	0.5 ± 0.04 ^a^	0.4 ± 0.03 ^b^	nd	Nd	nd	nd	0.02 ± <0.0001 ^c^	0.01 ± <0.0001 ^b^
Dimethyl sulphide	3.57	nd	nd	Nd	2.9 ± 0.04 ^a^	2.2 ± 0.03 ^b^	nd	Nd	nd	nd	0.7 ± 0.013 ^a^	0.6 ± 0.002 ^b^
Dimethyl disulphide	7.31	nd	nd	Nd	0.2 ± 0.004 ^a^	0.1 ± 0.004 ^b^	nd	Nd	nd	nd	0.03 ± <0.0001 ^a^	0.01 ± <0.0001 ^b^
1,2-butane-diol	7.74	nd	nd	Nd	0.1 ± 0.002 ^a^	0.06 ± 0.002 ^b^	nd	Nd	nd	nd	0.04 ± 0.002 ^c^	0.01 ± 0.002 ^d^
Phenyl ethyl alcohol	35.14	nd	nd	Nd	0.6 ± 0.04 ^a^	0.5 ± 0.03 ^b^	nd	Nd	0.02 ± <0.0001 ^f^	0.01 ± 0.003 ^g^	0.10 ± <0.0001 ^d^	0.06 ± <0.0001 ^e^
Butylated hydroxy toluene	34.86	nd	nd	Nd	0.3 ± 0.04 ^a^	0.2 ± 0.03 ^b^	0.1 ± 0.03 ^c^	0.07 ± 0.01 ^d^	nd	nd	0.2 ± 0.003 ^c^	0.09 ± 0.002 ^d^
2,3-butanedione	5.39	nd	nd	Nd	0.4 ± 0.004 ^a^	0.3 ± 0.004 ^b^	nd	nd	nd	nd	nd	nd
4-hydroxy-4methl-2-pentanone	16.57	nd	nd	Nd	0.02 ± 0.0024 ^a^	0.02 ± 0.0022 ^b^	nd	nd	nd	nd	0.001 ± <0.0001 ^c^	0.0009 ± <0.0001 ^d^

Peak areas are means of two GC–MS runs and approximated to one decimal place except when values are very low; nd = not detected. Different letters are significant differences between each packaged fillet; MAP − PAD: active-MA without absorbent pad; MAP + PAD: active-MA with absorbent pad, PMAP − PAD: passive-MA without absorbent pad, PMAP + PAD: passive-MA with absorbent pad and RT = retention time. Sampling was stopped on days when bacterial growth exceeded microbial limits < 5.5 log cfu/g by day 6 [38,39]; thus, sampling for fillets stored under PMAP at 4 °C was stopped on day 3. Furthermore, sampling for fillets stored under PMAP at 0 °C was stopped on day 6. Table S1 shows data for days 0, 3, 6, 9 and 12.

**Table 4:** Pearson’s correlation coefficient matrix between aerobic mesophilic counts selected spoilage quality indicators measured in Cape hake fillets during storage.

Variables	AMC	EA	BE	DMS	DMDS	TMA
AMC	**1**					
EA	**0.565**	**1**				
BE	**0.661**	**0.901**	**1**			
DMS	**0.677**	**0.887**	**0.910**	**1**		
DMDS	**0.728**	**0.825**	**0.908**	**0.968**	**1**	
TMA	**0.496**	**0.843**	**0.878**	**0.796**	**0.738**	**1**

Correlation values in **bold** are significant at *p* < 0.05. AMC = aerobic mesophillic count, EA = ethyl acetate, BE = butanoic acid ester, DMS= dimethyl sulphide, DMDS = dimethyl disulphide, TMA = tri-methyl amine.

## Data Availability

Data is contained within the article or Supplementary Materials.

## Supplementary Materials

The following supporting information can be downloaded at: https://www.mdpi.com/article/10.3390/foods11091292/s1, Table S1 Common volatiles identified from MA-packaged Cape hake fillets using gas chromatography–mass spectra analysis showing freshness and spoilage indicator markers expressed as % peak areas by sampling days 0, 3, 6, 9, and 12.
