# Supplementary material for: Changes in Volatile Composition of Cape Hake Fillets under Modified Atmosphere Packaging Systems during Cold Storage
Source: Foods. 2022 Apr 29;11(9):1292. doi: 10.3390/foods11091292 (PMC9103146; doi:10.3390/foods11091292)
Supplement: Supplementary file 1 [file foods-11-01292-s001.zip › foods-1660131-supplementary.pdf]

Table S1. Common volatiles identified from MA-packaged Cape hake fillets using gas chromatography–mass spectra analysis showing freshness and spoilage indicator markers expressed as % peak areas by sampling days 0, 3, 6, 9, and 12.

Common volatiles	RT (Min)	Day 0	Day 3							
			0 °C MAP – PAD	0 °C MAP + PAD	0 °C PMAP – PAD	0 °C PMAP + PAD	4 °C MAP – PAD	4 °C MAP + PAD	4 °C PMAP – PAD	4 °C PMAP + PAD
Ethyl alcohol	4.73	0.6 ± 0.10 [t]	3.3 ± 0.04 [h]	3.2 ± 0.01 [i]	4.9 ± 0.02 [a]	4.5 ± 0.02 [b]	3.5 ± 0.02 [f]	3.4 ± 0.01 [g]	3.8± 0.02 [c]	3.7± 0.02 [d]
3- methyl butanol	10.4	0.1 ± 0.03 [q]	0.15 ± 0.01 [o]	0.13 ± 0.01 [p]	0.72± 0.03 [e]	0.68± 0.03 f	0.33 ± 0.02 [k]	0.27 ± 0.02 [m]	0.54 ± 0.02 [g]	0.52 ± 0.02 [g]
2-ethyl hexanol	20.9	0.3 ± 0.06 [a]	nd	nd	0.04± 0.02 [b]	0.03± 0.01 [c]	nd	nd	nd	nd
3-octanone	12.36	0.2 ± 0.01 [s]	0.21 ± 0.041 [r]	0.21 ± 0.01 [r]	0.73± 0.01 [f]	0.64± 0.01 [h]	0.33 ± 0.01[o]	0.26 ± 0.01 [q]	0.55 ± 0.01 [i]	0.52 ± 0.01 [k]
Tri-methylamine	3.79	nd	nd	nd	4.3 ± 0.03 [e]	3.7 ± 0.02 [f]	nd	nd	5.1 ± 0.01 [c]	4.5 ± 0.01 [d]
Ethyl Acetate	4.35	nd	nd	nd	1.14± 0.05 [e]	0.86± 0.03 [f]	nd	nd	nd	nd
Butanoic acid Ester	6.32	nd	nd	nd	0.32± 0.002 [e]	0.26± 0.001 [f]	nd	nd	nd	nd
Acetic acid	19.92	nd	nd	nd	0.14± 0.003 [e]	0.12± 0.002 [f]	nd	nd	nd	nd
3-methyl thio-1-Propanol	29.71	nd	nd	nd	0.04± 0.003 [e]	0.03± 0.002 [f]	nd	nd	nd	nd
Dimethyl sulphide	3.57	nd	nd	nd	0.2 ± 0.003 [e]	0.1 ± 0.002 [f]	nd	nd	nd	nd
Dimethyl disulphide	7.31	nd	nd	nd	nd	nd	nd	nd	nd	nd
1,2-Butane-diol	7.74	nd	nd	nd	nd	nd	nd	nd	nd	nd
Phenyl ethyl Alcohol	35.14	nd	nd	nd	0.2 ± 0.003[c]	0.1 ± 0.002 [d]	nd	nd	nd	nd
Butylated hydroxy toluene	34.86	nd	nd	nd	0.02± 0.003[f]	0.02± 0.002 [f]	nd	nd	nd	nd
2,3- butanedione	5.39	nd	nd	nd	0.01± 0.001c	0.01± 0.001 [d]	nd	nd	nd	nd
4-hydroxy-4methl-2-penta-none	16.57	nd	nd	nd	nd	nd	nd	nd	nd	nd

Peak areas are means of two GC-MS runs and approximated to one decimal places except when values are very low; nd = not detected. Different letters are significant differences between each packaged fillet; MAP - PAD: active-MA without absorbent pad; MAP + PAD: active-MA with absorbent pad, PMAP - PAD: passive-MA without absorbent pad, PMAP + PAD: passive-MA with absorbent pad and RT = retention time.
Sampling was stopped on days when bacterial growth exceeded microbial limits < 5.5 log cfu/g by day 6 (DOH, 2001; HPA, 2009), thus, sampling for fillets stored under PMAP at 4°C was stopped on day 3, furthermore, sampling for fillets stored under PMAP at 0°C was stopped on day 6.

Table S1. (Continuation)

Common volatiles	RT (Min)	Day 6					
		0 °C MAP – PAD	0 °C MAP + PAD	0 °C PMAP – PAD	0 °C PMAP + PAD	4 °C MAP – PAD	4 °C MAP + PAD
Ethyl alcohol	4.73	2.3 ± 0.12 m	2.2 ± 0.12 n	3.6± 0.12 e	3.5± 0.12 f	3.0 ± 0.12 j	2.9 ± 0.12 k
3- methyl butanol	10.4	0.3 ± 0.04 l	0.2 ± 0.01 n	1.0± 0.03 c	0.9± 0.02 d	0.5 ± 0.01 h	0.5 ± 0.01 h
2-ethyl hexanol	20.9	nd	nd	0.2± 0.02 b	0.2± 0.02 c	nd	nd
3-octanone	12.36	0.4± 0.01 n	0.3 ± 0.02 p	1.0± 0.02 a	0.9± 0.02 b	0.5 ± 0.02 l	0.4 ± 0.01 n
Tri-methylamine	3.79	nd	nd	7.2± 0.07 a	5.4± 0.05 b	nd	nd
Ethyl Acetate	4.35	nd	nd	3.3± 0.01 a	3.1± 0.02 b	nd	nd
Butanoic acid Ester	6.32	nd	nd	0.78 ± 0.002 a	0.59± 0.001 b	nd	nd
Acetic acid	19.92	nd	nd	0.4± 0.002 a	0.4± 0.002 b	nd	nd
3-methyl thio-1-Propanol	29.71	nd	nd	0.5± 0.04 a	0.4± 0.03 b	nd	nd
Dimethyl sulphide	3.57	nd	nd	2.9± 0.04 a	2.2± 0.03 b	nd	nd
Dimethyl disulphide	7.31	nd	nd	0.2± 0.004 a	0.1± 0.004 b	nd	nd
1,2-Butane-diol	7.74	nd	nd	0.1± 0.002 a	0.06± 0.002 b	nd	nd
Phenyl ethyl Alcohol	35.14	nd	nd	0.6± 0.04 a	0.5± 0.03 b	nd	nd
Butylated hydroxy toluene	34.86	nd	nd	0.3 ± 0.04 a	0.2 ± 0.03 b	0.1 ± 0.03 c	0.07 ± 0.01 d
2,3- butanedione	5.39	nd	nd	0.4± 0.004 a	0.3± 0.004 b	nd	nd
4-hydroxy-4methl-2-pentanone	16.57	nd	nd	0.02± 0.0024 a	0.02± 0.0022 b	nd	nd

Peak areas are means of two GC-MS runs and approximated to one decimal places except when values are very low; nd = not detected. Different letters are significant differences between each packaged fillet; MAP - PAD: active-MA without absorbent pad; MAP + PAD: active-MA with absorbent pad, PMAP - PAD: passive-MA without absorbent pad, PMAP + PAD: passive-MA with absorbent pad and RT = retention time.
Sampling was stopped on days when bacterial growth exceeded microbial limits < 5.5 log cfu/g by day 6 (DOH, 2001; HPA, 2009), thus, sampling for fillets stored under PMAP at 4°C was stopped on day 3, furthermore, sampling for fillets stored under PMAP at 0°C was stopped on day 6.

Table S1. (Continuation)

Common volatiles	RT (Min)	Day 9				Day 12			
		0 °C MAP – PAD	0 °C MAP + PAD	4 °C MAP – PAD	4 °C MAP + PAD	0 °C MAP – PAD	0 °C MAP + PAD	4 °C MAP – PAD	4 °C MAP + PAD
Ethyl alcohol	4.73	1.9 ± 0.02 ^{p}	1.8 ± 0.01 ^{q}	2.9 ± 0.12 ^{k}	2.8 ± 0.12 ^{l}	1.1 ± 0.12 ^{qr}	0.7 ± 0.12 ^{s}	2.2 ± 0.12 ^{n}	2.1 ± 0.12 ^{o}
3- methyl butanol	10.4	0.41 ± 0.02 ^{i}	0.36 ± 0.02 ^{j}	0.54 ± 0.02 ^{g}	0.50 ± 0.02 ^{h}	0.7 ± 0.01 ^{f}	0.5 ± 0.01 ^{h}	1.3 ± 0.03 ^{a}	1.1 ± 0.01 ^{b}
2-ethyl hexanol	20.9	nd	nd	nd	nd	nd	nd	nd	nd
3-octanone	12.36	0.54 ± 0.02 ^{j}	0.45 ± 0.01 ^{m}	0.76 ± 0.01 ^{e}	0.69 ± 0.01 ^{g}	0.8 ± 0.01 ^{d}	0.55 ± 0.02 ^{i}	0.88 ± 0.01 ^{c}	0.8 ± 0.02 ^{d}
Tri-methylamine	3.79	nd	nd	1.9 ± 0.03 ^{i}	1.5 ± 0.02 ^{j}	0.3 ± 0.03 ^{k}	0.1 ± 0.03 ^{l}	2.4 ± 0.14 ^{g}	1.8 ± 0.13 ^{h}
Ethyl Acetate	4.35	nd	nd	0.52 ± 0.03 ^{g}	0.46 ± 0.03 ^{h}	nd	nd	1.2 ± 0.01 ^{c}	1.2 ± 0.01 ^{d}
Butanoic acid Ester	6.32	nd	nd	0.15 ± 0.002 ^{g}	0.09 ± 0.001 ^{h}	nd	nd	0.27 ± 0.001 ^{c}	0.24 ± 0.001 ^{d}
Acetic acid	19.92	nd	nd	nd	nd	nd	nd	0.2± 0.007 ^{c}	0.1 ± 0.002 ^{d}
3-methyl thio-1-Pro-panol	29.71	nd	nd	nd	nd	nd	nd	0.02 ± 0.0004 ^{c}	0.01 ± 0.0004 ^{d}
Dimethyl sulphide	3.57	nd	nd	nd	nd	nd	nd	0.7 ± 0.013 ^{c}	0.6 ± 0.002 ^{d}
Dimethyl disulphide	7.31	nd	nd	nd	nd	nd	nd	0.03 ± 0.0004 ^{a}	0.01 ± 0.0004 ^{b}
1,2-Butane-diol	7.74	nd	nd	nd	nd	nd	nd	0.04± 0.002 ^{c}	0.01 ± 0.002 ^{d}
Phenyl ethyl Alco-hol	35.14	nd	nd	nd	nd	0.02 ± 0.0004^{f}	0.01 ± 0.0003^{g}	0.10 ± 0.0003 ^{d}	0.06 ± 0.0002 ^{e}
Butylated hydroxy toluene	34.86	nd	nd	nd	nd	nd	nd	0.2 ± 0.003 ^{b}	0.09 ± 0.002 ^{d}
2,3- butanedione	5.39	nd	nd	nd	nd	nd	nd	nd	nd
4-hydroxy-4methl-2-pentanone	16.57	nd	nd	nd	nd	nd	nd	0.001± 0.0003 ^{c}	0.00009± 0.00001 ^{d}

Peak areas are means of two GC-MS runs and approximated to one decimal places except when values are very low; nd = not detected. Different letters are significant differences between each packaged fillet; MAP - PAD: active-MA without absorbent pad; MAP + PAD: active-MA with absorbent pad, PMAP - PAD: passive-MA without absorbent pad, PMAP + PAD: passive-MA with absorbent pad and RT = retention time.
Sampling was stopped on days when bacterial growth exceeded microbial limits < 5.5 log cfu/g by day 6 (DOH, 2001; HPA, 2009), thus, sampling for fillets stored under PMAP at 4°C was stopped on day 3, furthermore, sampling for fillets stored under PMAP at 0°C was stopped on day 6.
